# Evaluating the impact of continuing professional development courses on physician behavioral intention: a pre-post study with follow-up at six months

**DOI:** 10.1186/s12909-023-04597-3

**Published:** 2023-09-03

**Authors:** Felly Bakwa Kanyinga, Amédé Gogovor, Suélène Georgina Dofara, Souleymane Gadio, Martin Tremblay, Sam J. Daniel, Louis-Paul Rivest, France Légaré

**Affiliations:** 1 Department of Social and Preventive Medicine, Faculty of Medicine, Université Laval, Quebec City, QC G1J 2G1 Canada; 2 VITAM — Centre de recherche en santé durable, Centre intégré universitaire de santé et de services sociaux de la Capitale-Nationale, 2480 Chemin de la Canardière, Quebec City, QC G1J 2G1 Canada; 3 Department of Family Medicine and Emergency Medicine, Faculty of Medicine, Université Laval, Quebec City, QC G1J 2G1 Canada; 4 Continuing Professional Development Directorate, Fédération des Médecins Spécialistes du Québec, Montreal, QC H5B 1G8 Canada; 5 Department of Mathematics and Statistics, Faculty of Science and Engineering, Université Laval, Quebec City, QC G1V 0A6 Canada

**Keywords:** Continuing professional development, Intention, Course, Behavior, Physician

## Abstract

**Background:**

Continuing professional development (CPD) for health professionals includes educational activities to maintain or improve skills. We evaluated the impact of a series of CPD courses by identifying factors influencing physicians’ intention to adopt targeted behaviors and assessing self-reported behavior adoption six months later.

**Methods:**

In this pre-post study, eligible participants attended at least one in-person course at the Fédération des Médecins Spécialistes du Québec annual meeting in November 2019. Before and afterwards, participants completed CPD-REACTION, a validated questionnaire based on Godin’s integrated model for health professional behavior change that measures intention and psychosocial factors influencing intention. We used Wilcoxon signed-rank test to compare pre- and post-course intention scores and linear regression analyses to identify factors influencing intention. We also compared the post-course intention scores of participants reporting a behavior change six months later with the scores of those reporting no behavior change six months later. Qualitative data was collected only six months after courses and responses to open-ended questions were analyzed using the Theoretical Domains Framework.

**Results:**

A total of 205/329 course attendees completed CPD-REACTION (response rate 62.3%). Among these participants, 158/329 (48%) completed the questionnaire before CPD courses, 129/329 (39.2%) only after courses and 47/329 (14.3%) at 6 months. Study population included 192 physicians of whom 78/192(40.6%) were female; 59/192(30.7%) were between 50 and 59 years old; and 72/192 (37.5%) were surgical specialist physicians. Mean intention scores before (n = 158) and after (n = 129) courses were 5.74(SD = 1.52) and 6.35(SD = 0.93) respectively. Differences in mean (DM) intention before and afterwards ranged from − 0.31(p = 0.17) to 2.25(p = 0.50). Multivariate analysis showed that beliefs about capabilities (β = 0.15, p = 0.001), moral norm (β = 0.75, p < 0.0001), and beliefs about consequences (β = 0.11, p = 0.04) influenced post-course intention. Post-course intention was correlated with behavior six months later (DM = 0.63; p = 0.02). Qualitative analysis showed that facilitators to behavior adoption after six months were most often related to the TDF domains of beliefs about capabilities. Most frequent barriers to adoption related to lack of resources.

**Conclusions:**

Overall, scores for intention to adopt targeted behaviors increased after the courses. CPD providers could increase participants’ intention by including interventions that emphasize beliefs about capabilities, moral norm and beliefs about consequences.

**Supplementary Information:**

The online version contains supplementary material available at 10.1186/s12909-023-04597-3.

## Background

Continuing professional development (CPD) updates physicians’ knowledge and skills with the aim of transferring new knowledge into practice [1–4] and may improve clinical outcomes [5, 6]. CPD courses are often based on Kirkpatrick’s framework and Bloom’s taxonomy, which categorize learning objectives into various domains and levels of complexity [7, 8]. However, few CPD designers use socio-cognitive theories to target factors shown to influence behavior change, [9–11] and CPD providers rarely evaluate courses using validated tools [12–15].

Informed by the Theory of Planned Behavior (TPB), Godin’s integrated behavior change framework for health professionals is based on a systematic review of 76 studies, and assumes that intention influences the behavior of health professionals [15]. Intention in turn is influenced by their characteristics as well as by four modifiable psychosocial factors: beliefs about capabilities, moral norm, social influences and beliefs about consequences [15]. The systematic review led to the development of the CPD-REACTION questionnaire, a valid and reliable tool based on socio-cognitive theories for evaluating the impact of CPD activities on health professionals’ intention to adopt targeted behaviors. The questionnaire measures intention and the psychosocial factors that influence intention [4, 16, 17]. Studies examining the associations between these factors and intention have showed that high intention scores for behavior change, regardless of the measurement tool used, do not necessarily ensure the occurrence of actual behavior change [15, 18, 19]. These studies suggest that moderate to high levels of intention are generally linked to modest to moderate changes in behavior. In other words, while many participants may indicate the intention to adopt a particular behavior, not all of them will ultimately follow adopt it [20–23]. Moreover, few studies conducted with health professionals have followed up to see whether the behavior was adopted or had any effect on patient outcomes [18, 19, 24, 25]. In view of these gaps, this study aimed to evaluate the impact of a series of CPD courses for specialist physicians by identifying factors influencing their intention to adopt targeted behaviors and also assessing adoption of the behavior six months later [26].

## Methods

### Study design and setting

We performed a pre-post study to evaluate the impact of a series of CPD courses for specialist physicians by identifying factors influencing their intention to adopt targeted behaviors and assessing adoption of the behavior six months later. The CPD courses were given during a Fédération des Médecins Spécialistes du Québec (FMSQ) in-person annual meeting. We reported data according to the STROBE reporting guidelines for observational studies [27].

### Participants and recruitment

On November 15, 2019, the FMSQ held its annual meeting in Quebec City, Canada. The FMSQ represents 59 medical specialties and has a membership of more than 10,000.

Inclusion criterion for participants in this study were to be a physician who attended one or more of nine selected courses.

Physicians who attended at least one selected course were eligible for the study. Physicians who did not complete the CPD-REACTION questionnaire at any time were excluded.

### Intervention

At the FMSQ meeting the CPD courses included in our study were offered as in-person didactic lectures that incorporated additional supporting techniques such as slide presentations, multimedia presentations and presentations by medical experts. The CPD courses were accredited by the Royal College of Physicians and Surgeons of Canada [28]. Courses were eligible for our study if: (1) the scientific committee of each course agreed to include it; (2) it defined a main behavior change; (3) it targeted one of the seven CanMEDS roles [29]. For more details see Appendix 1.

### Data collection and variables

Data were collected anonymously from the participants between November 2019 and June 2020. One week before the meeting, physicians registered were invited to complete (1) a sociodemographic questionnaire and (2) the CPD-REACTION questionnaire [17] for each CPD course they planned to attend. Reminders were sent just before courses began. Immediately afterwards, participants were again invited to complete CPD-REACTION and, six months after the meeting, invited to complete a self-reported behavior change questionnaire. A reminder was automatically sent two weeks later. All questionnaires were self-administered online on the FMSQ web-based interactive platform (MÉDUSE).

#### Dependent variable

The intention to adopt a behavior after the courses, our main outcome, was evaluated using CPD-REACTION, a validated tool [4, 16, 17]. Twelve items evaluate intention and factors associated with it [16]. Scores are the mean of each construct item measured using a Likert-type scale from 1 (low) to 7 (high) [16]. The Cronbach’s alpha coefficient ranges from 0.77 to 0.85 [17]. The CPD-REACTION questionnaires were adapted to the behavior change targets of each course [16].

#### Independent variables

According to Godin’s integrated behavior change framework, the factors influencing intention are beliefs about capabilities (three items), social influences (three items), moral norm (two items) and beliefs about consequences (two items) [16]. Beliefs about capabilities reflect confidence or self-efficacy about adopting the behavior. Social influences are the perception of approval by people important to the participant. Moral norm is the feeling of personal obligation to adopt the behavior according to personal values. Beliefs about consequences is the perception of the usefulness and benefits of adopting the behavior [30].

Participants provided sociodemographic characteristics that included age, sex (female or male) and their medical association (clinical area).

#### Other variables

Six months after the courses, participants completed a self-reported behavior change questionnaire about (1) whether they had adopted the targeted behavior(s) (yes/no), and whether (2) they had made any other change(s) in their professional practice following the courses (yes/no), 2a) if so, how, 2b) if not, why not, (3) whether the CPD course had any impact on patient safety or health (outcomes), 3a) if so, what impact, and 3b) if not, why not. Questions 2a) to 3b) were open-ended and provided space for respondents to elaborate.

### Analysis

We used Godin’s integrated behavior change framework for health professionals [15] for quantitative analysis and the Theoretical Domains Framework (TDF) [31, 32] for qualitative analysis.

Generally, research surveys conducted among physicians are characterized by a low response rate [33, 34]. Combining the courses gave us an adequate sample size, i.e., statistical power for the quantitative analyses, and also allowed us to evaluate the impact of the annual CPD meeting as a whole.

#### Quantitative analysis

We performed descriptive statistics for all variables. To compare pre- and post-intervention scores for each course and all courses together, we used the Wilcoxon signed rank test as the normality assumption was rejected. Since our samples were small and since many participants had the same pre- and post-course scores, the confidence intervals associated with this test, based on pseudo medians, are unreliable. Therefore, only p-values were used to determine whether courses had a significant influence on intention. We considered a course cohort as a fixed effect [35].

As yet there is no benchmark score that signals a statistically significant higher intention. Studies on the validation of the CPD-REACTION questionnaire showed that the highest scores are higher than the mean of the range ((7-0)/2=3,5) and tend to be closer to the maximum score, which is 7 [4, 16]. The comparison analysis of these means helped highlight a statistically significant difference between the scores for a better interpretation of the data.

We performed ANOVA single-factor and two-factor (considering time) analyses to compare intention between courses. Exploratory analyses were performed to estimate the intraclass coefficient (ICC) for evaluating the percentage of variance in intention and in its psychosocial factors that were attributable to the CPD courses. We also performed Spearman correlations analysis to evaluate the association of each independent continuous variable with physicians’ post-course intention. We used linear regression analysis to perform bivariate analyses to examine the relationship between intention scores and each independent variable (at alpha level < 0.20). Then we performed multivariate regression analysis using a manual backward stepwise selection of the variables with a significance level (p-value) of 0.05. After obtaining the final model, the variables that had been excluded during selection were reintroduced one by one into the model to check if their presence improved it. We considered p-values of < 0.05 as statistically significant in the final (predictive) model. To assess study robustness, we performed one sensitivity analysis that excluded participants who attended two courses and therefore had two measures of post-course intention; and another sensitivity analysis with physicians who completed CPD-REACTION both before and after courses. Finally, using the Wilcoxon test, we compared post-course intention scores among participants who reported having adopted the targeted behavior six months later with post-course intention scores of those who reported not having adopted the targeted behavior.

As courses were conducted before the study began, participants were already recruited so the sample size could not be changed. We therefore performed a post-hoc power calculation for our study. Based on another study that used CPD-REACTION with a single category of health professionals, a sample size of 60 participants is required to detect a difference in means (DM) of 0.44 (1.2) between intention measured before and after courses (considering a significance level (α) of 5% and approximate power of 80%) [36]. To complete the power calculation we calculated the standardized effect size for a DM from an estimation formula informed by the literature [37].

We used Statistical Analysis Software (SAS) version 9.4 (SAS Institute Inc., Cary, NC, USA) and RStudio software version Desktop 2022.07.0.

#### Qualitative analysis

The qualitative analysis aimed to better capture the views of the participants and to further explore the reasons for their post-course intention scores. We used the TDF to summarize and aggregate the qualitative data because (a) the TDF has a strong empirical basis and provides a method for theoretically assessing implementation problems, as well as professional and other health-related behaviors, for the purpose of intervention development; (b) the TDF facilitates identification of the determinants of a given behavior to generate well-structured and concise summaries of the collected data [38, 39]. Two researchers (F.B.K., A.G.) with different levels of experience in qualitative analysis independently analyzed, reviewed and agreed on answer codes for the responses to the open-ended questions collected six months later. Data were coded using a thematic deductive approach and refined into TDF domains [32]. French transcripts were translated (F.B.K.) and reviewed by a scientific translator. We calculated the frequency of barriers and facilitators found in each TDF domain.

## Results

### Characteristics of participants

**Fig. 1:**
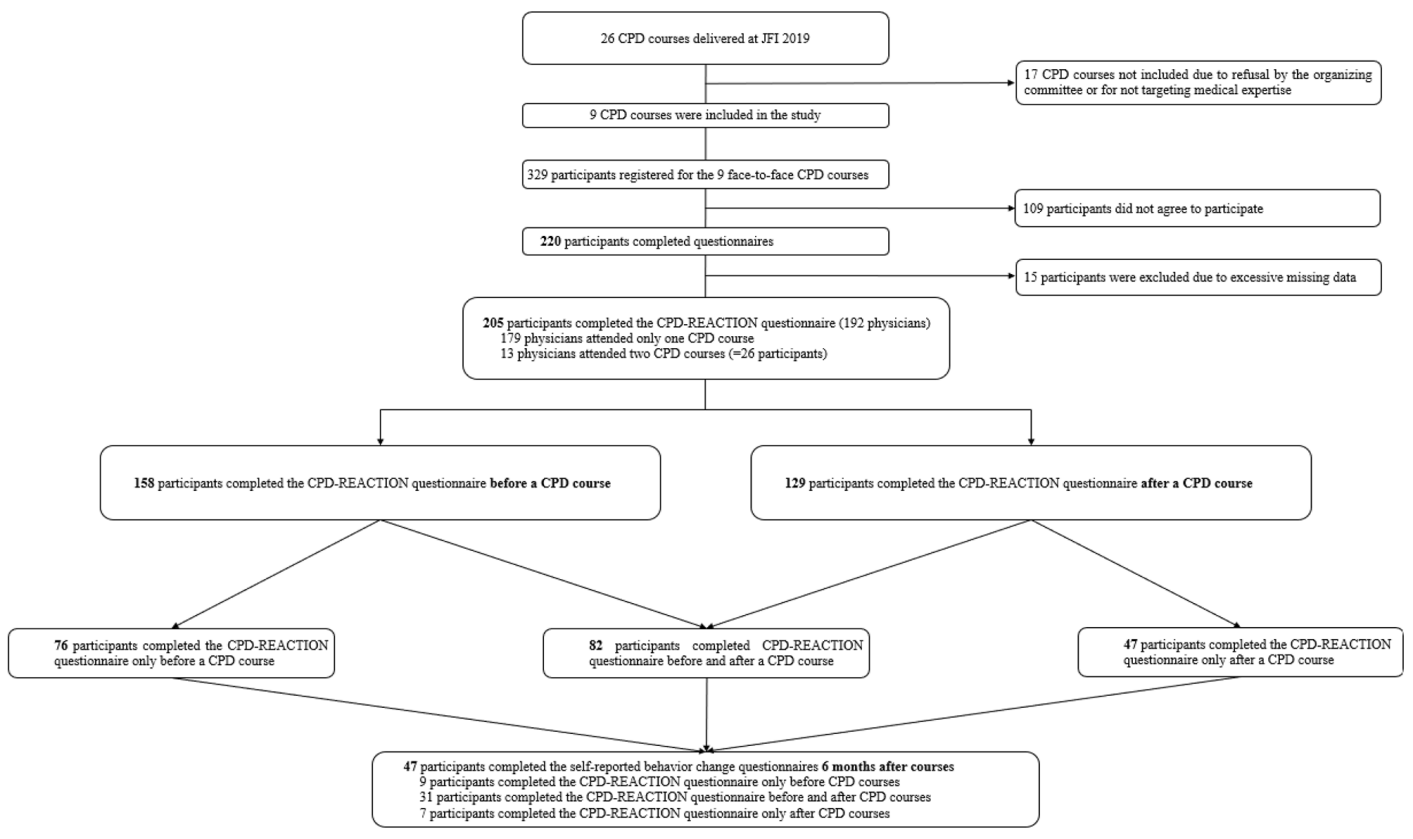
Flow chart of the study

Of the 329 participants who attended the selected nine courses, 205 completed the CPD-REACTION questionnaire, representing a 62.3% response rate. More specifically, 158/329 (48%) completed CPD-REACTION before courses, 128/329 (39.2%) after the courses and 47/329 (14.3%) at 6 months. Of the 26 potentially eligible courses, 9/26 (34.6%) were included (Fig. 1). The 13 physicians who had taken two of the nine courses each counted as two participants. Among the physicians, 78/192 (40.6%) were female, 59/192 (30.7%) were between 50 and 59 years old and 72/192 (37.5%) practiced in surgical specialties (Table 1).

**Table 1:** Characteristics of physicians

	Number (n) Total = 192	Percentage (%) Total = 100%
Socio-demographic characteristics of physicians		
Age (in years)		
30 — 39	24	12.5
40 — 49	35	18.2
50 — 59	59	30.7
> = 60	35	18.2
Missing	39	20.3
Sex		
Female	78	40.6
Male	75	39.1
Missing	39	20.3
Profession		
Medical specialist	153	79.7
Missing	39	20.3
Medical specialty		
Surgical		
Anesthesiology	22	11.5
Surgery	8	4.2
Neurosurgery	2	1.0
Obstetrics and Gynecology	6	3.1
Ophtalmology	2	1.0
Orthopedic Surgery	23	12.0
Otolaryngology – Head and Neck Surgery	5	2.6
Plastic Surgery	4	2.1
Laboratory		
Hematological Pathology and Oncology	6	3.1
Medicine – Biochemistry	2	1.0
Medicine microbiology and Infectiology	1	0.5
Nuclear medicine	4	2.1
Pathology	1	0.5
Diagnostic Radiology	10	5.2
Medical		
Cardiology	8	4.2
Geriatrics	1	0.5
Psychiatry	12	6.3
Dermatology	1	0.5
Neurology	1	0.5
Physiatry	7	3.7
Respirology	1	0.5
Pediatrics	14	7.3
Emergency Medicine	2	1.0
Internal Medicine	5	2.6
Public Health and Preventive Medicine	3	1.6
Other	2	1.0
Missing	39	20.3

### Behavioral intention

#### Evaluated either before or after courses

For those who completed CPD-REACTION before courses (n = 158), mean intention scores out of 7 were 5.7 (SD = 1.52); and for those who completed CPD-REACTION after courses (n = 129), mean intention scores were 6.35 (SD = 0.93) (Table 2).

**Table 2:** Intention and Psychosocial Factors Influencing Intention

		Before CPD courses			After CPD courses		
CPD-REACTION constructs		n	Mean (SD)	Median (Interquartile range)	n	Mean (SD)	Median (Interquartile range)
	Intention	157	5.74 (1.52)	6.00 (5.00;7.00)	129	6.35 (0.93)	7.00 (6.00;7.00)
	Beliefs about capabilities	157	5.05 (1.45)	5.33 (4.33;6.00)	129	5.90 (1.02)	6.00 (5.67;6.67)
	Moral norm	158	5.99 (1.18)	6.00 (5.50;7.00)	127	6.48 (0.85)	7.00 (6.00;7.00)
	Social influences	158	4.09 (1.35)	4.33 (3.00;5.33)	129	4.61 (1.20)	5.00 (4.00;5.33)
	Beliefs about consequences	158	5.91 (1.29)	6.00 (5.00;7.00)	129	6.31 (0.98)	7.00 (6.00;7.00)
Abbreviations: CPD indicates continuing professional development; SD indicates Standard deviation							

#### Compared before and after courses

Intention scores among participants who completed CPD-REACTION both before and after courses (n = 81) were 5.99 (SD = 1.31) and 6.44 (SD = 0.80) respectively, a significant mean difference of 0.45 (p = 0.002). Mean difference in before/after intention varied across individual courses from − 0.31 (p = 0.17) to 2.25 (p = 0.50). The difference was significant only for the course on sports injuries 0.60 (p = 0.01) (Table 3).

**Table 3:** Comparison of behavioral intention evaluated both before and after CPD courses

CPD courses	Mean score of the intention before CPD course		Mean score of the intention after CPD course		Mean difference	
	n	Mean (SD)	n	Mean (SD)	Difference	p-value^a^
**All 9 CPD** courses	81	5.99 (1.31)	81	6.44 (0.80)	0.45	**0.002**
**By CPD** course						
CPD course 1 — Patient safety	6	6.75 (0.42)	6	6.75 (0.42)	0.00	1.00
CPD course 2 — Care incidents	4	5.38 (2.14)	4	6.75 (0.50)	1.38	0.37
CPD course 3 — Optimization of care	2	4.75 (0.35)	2	7.00 (0.00)	2.25	0.50
CPD course 4 — Perioperative pain and opioids	8	6.81 (0.37)	8	6.50 (0.60)	-0.31	0.17
CPD course 5 — Sports injuries	24	5.25 (1.38)	24	5.85 (1.02)	0.60	**0.01**
CPD course 6 — Eating disorders	9	6.33 (1.12)	9	6.94 (0.17)	0.61	0.11
CPD course 7 — Attention deficit and hyperactivity disorder	6	5.92 (1.02)	6	6.67 (0.52)	0.75	0.17
CPD course 8 — Cardio-oncology	9	6.44 (0.73)	9	6.44 (0.53)	0.00	1.00
CPD course 9 — Local anesthesia	13	6.35 (1.49)	13	6.69 (0.72)	0.35	0.58

Abbreviations: CPD indicates continuing professional development; SD indicates standard deviation

^a^: p-value significant at < 0.05 — Obtained using the Wilcoxon signed rank test

p-value in bold: significant

#### Variation between courses and ICC estimates

Analysis of variance (ANOVA) showed significant variation of intention, both between course cohorts pre-courses (F value = 3.75; p = 0.0005) and before/after courses (F value = 2.57; p = 0.01). However, intention after courses did not vary significantly across courses (F value = 0.73; p = 0.67) (ANOVA variance analysis data not shown). On an exploratory basis, we performed ICC estimates for intention and each of its psychosocial determinants before and after the courses. We observed ICC estimates for intention of 0.14 before (Appendix 2) and 0.004 after the courses (Appendix 3).

### Factors associated with intention

Bivariate regression analysis of sociodemographic and psychosocial factors showed that six variables were associated with intention at a threshold of p-value < 0.20 (Table 4). After manual backward stepwise selection variables, only moral norm, beliefs about capabilities and beliefs about consequences stayed for the final multivariate regression analysis (n = 129), which showed that moral norm (β = 0.75, p < 0.0001), beliefs about capabilities (β = 0.15, p = 0.001) and beliefs about consequences (β = 0.11, p = 0.04) influenced physicians’ intention to adopt a behavior (Table 4). These factors explained about 82% of the variance of intention in the model. In sensitivity analysis, only moral norm (β = 0.75, p < 0.0001) and beliefs about capabilities (β = 0.15, p < 0.0003) remained significant after excluding the 13 physicians who had attended two courses (n = 111) (data not shown). Similarly, sensitivity analysis performed with physicians who completed CPD-REACTION both pre- and post-course (n = 81) showed that moral norm (β = 0.67, p < 0.0001) and beliefs about capabilities (β = 0.17, p < 0.0002) influenced intention (data not shown). Both sensitivity analyses showed that these two factors explained about 80% of the variance of intention after courses.

**Table 4:** Factors associated with physicians’ intention to adopt a behavior

Variables		n	β	CI 95%	p-value^a^
Bivariate regression analysis					
Age		88			**0.08**
	30–39 (vs. > = 60)		0.26	0.02 ; 1.07	0.04
	40–49 (vs. > = 60)		0.24	-0.07 ; 0.88	0.09
	50–59 (vs. > = 60)		0.21	-0.36 ; 0.46	0.82
Sex		88			**0.002**
	Female (vs. Male)		0.50	0.19; 0.81	0.002
Medical specialty category		88			0.76
	Surgical (vs. Clinic)		-0.06	-0.42; 0.33	0.75
	Laboratory (vs. Clinic)		-0.19	-0.70; 0.32	0.46
Beliefs about capabilities		129	0.62	0.50; 0.72	**< 0.0001**
Social influences		129	0.32	0.19 ; 0.44	**< 0.0001**
Moral norm		127	0.95	0.87; 1.04	**< 0.0001**
Beliefs about consequences		129	0.65	0.53; 0.77	**< 0.0001**
**Multivariate regression analysis (R** ^**2**^ **adj = 0.82)**					
Beliefs about capabilities		127	0.15	0.06; 0.24	**0.001**
Moral norm		127	0.75	0.62; 0.88	**< 0.0001**
Beliefs about consequences		127	0.11	0.01; 0.21	**0.04**

Abbreviations: β indicates the coefficient; CI 95% indicates confidence interval at 95%; vs. indicates versus; R^2^ adj indicates adjusted R^2^

^a^: p-value significant at < 0.20 for bivariate regression analysis and at < 0.05 for multivariate regression analysis

p-value in bold: significant.

### Intention post-course in relation to reported behavior change at six months

Among the 47 physicians who completed the self-reported behavior change questionnaire six months later, 38 had completed CPD-REACTION immediately post-course. Of these 38, 31/38 (81.6%) participants reported adopting the behavior targeted by the course they had attended, and 7/38 (18.4%) reported not adopting it. Mean intention of the 31 participants who reported they had adopted the behavior was 6.63 (SD = 0.69), while intention of the seven participants who reported not adopting it was 6.00 (SD = 0.96), a statistically significant difference of 0.63 (p = 0.02) (data not shown).

### Qualitative findings

#### Barriers and facilitators mapped to the TDF domains

Regarding behavior changes six months after the course, barriers to adopting the behavior most frequently related to the TDF domains of environmental context and resources (e.g., not having enough time or not having the relevant technologies in the hospital); while facilitators most frequently mapped to the domains of both skills and beliefs about capabilities (e.g. for achieving better diagnosis and patient management). See Table 5. Regarding impact of courses on patient safety and health outcomes six months later, barriers most frequently related to the TDF domains of environmental context and resources (e.g. limited to the use of available material) and facilitators most related to skills (e.g. improved patient management). See Table 6.

**Table 5:** Barriers and facilitators (Theoretical Domains Framework) regarding adoption of behaviors

Theoretical domain	Barrier/ Facilitator	Representative excerpts of quotations^b^	Frequencies^c^
**Knowledge**	Recognition of the importance of involving patients’ families (Facilitator)	“Give more family appointments …”	2
	Better knowledge for their practice (Facilitator)	“Better knowledge of radiological techniques and their applications”	2
	Course is not new (Barrier)	“I was already familiar with the literature on the subject before”	2
	Acquisition of knowledge to strengthen competence (Facilitator)	“…, the cardio-oncology course served as a refresher on the interpretation of cardiac biopsies in oncology patients”	1
	More evidence-based research (Facilitator)	“Based on my reading of what was being done elsewhere”	1
**Skills**	Behavior already in place (Barrier)	“It was already in my practice”	3
	Better clinical assessment (Facilitator)	“… better self-criticism of cases seen in clinic”	1
	Expanded scope of practice (Facilitator)	“More cardio consultation …”	2
	Ensure better patient engagement (Facilitator)	“More asking the patient about his expectations”	1
	Better patient management (Facilitator)	“More aggressive search for coronary disease in patients with a history of thoracic radiotherapy”	3
	Adoption of a new protocol for hospitalizations (Facilitator)	“Protocol during hospitalization”	2
	Better use of imaging (Facilitator)	“Imaging a little earlier”	1
	Improved test ordering (Facilitator)	“More targeted TTE requests vs. isotopic ventricles”	1
	Improved prescription of procedural drugs (Facilitator)	“Adjust the dose of my local anesthetics”	2
	Improved perioperative preparation (Facilitator)	“Optimize pre-op preparation and analgesia for an ERC”	1
**Social/Professional Role And Identity**	Knowledge transfer to colleagues (Facilitator)	“Training my colleagues …”	1
	Better prescribing and referral to colleagues (Facilitator)	“Prescription of psychostimulants with advice to colleagues when patient with history of stroke”	1
	Better collaboration with colleagues from another medical specialty (Facilitator)	“Much closer collaboration with our radiologists”	3
**Beliefs About Capabilities**	Good understanding of the subject (Facilitator)	“Better information for the patient and good understanding of the re in children”	1
	Better management of patients (Facilitator)	“Better monitoring of patients on psychostimulants”	6
	Continuation of this known practice (Facilitator)	“It was already in my way of practice”	2
	Search for information (Facilitator)	“More reading and better self-criticism of cases seen in clinic”	1
	Validation of knowledge already acquired (Facilitator)	“I was already familiar with the literature on the subject before”	1
	Confidence in handling patients (Facilitator)	“More confident to prescribe psychostimulants even in a cardiac patient, with a supporting EKG”	2
	Better patient follow-up (Facilitator)	“More rigorous medical follow-up”	1
	Validation of an already well known practice (Facilitator)	“… already highly trained in radiological reading”	1
	Validation of an already well known approach (Facilitator)	“I already favored the clinicoradiological approach”	1
**Goals**	Better patient education (Facilitator)	“Inform patients with narcotics that it is dangerous to use them chronically”	1
	Better explanation to patients (Facilitator)	“I now take more time to explain the post-treatment consent recommendations”	1
	Better critique of the procedure (Facilitator)	“Ask more questions about TCAs”	1
	Rechecking of radiology exams (Facilitator)	“Review my x-rays”	1
**Environmental Context And** **Resources**	Lack of clientele for practice (Barrier)	“The opportunity did not present itself”	2
	Integration of new components into practice (Facilitator)	“Training my colleagues, preparation of a LAST kit”	1
	Lack of material resources for practice (Barrier)	“Ultrasound not available in orthopedics … ”	1
	Material resources not available (Barrier)	“Radiological modalities not available in my area”	1
	Maintenance of usual routine (Barrier)	“I have not incorporated cannabinoid prescribing into my practice. We use the saturometer during minor surgeries”	2
	Lack of time (Barrier)	“Haven’t had time to set up a cardio-oncology service corridor yet”	1
**Behavioral Regulation**	Unsuitable clientele (Barrier)	“I changed my clientele”	2
	Search for additional information (Facilitator)	“I have been researching different companies offering PRP for gonarthrosis”	2
	Adopted a new approach to follow-up (Facilitator)	“Modification to my method of tracking test results”	1
	Adoption of a different approach to managing adverse events (Facilitator)	“Different management in the disclosure of medical errors and management of sentinel events”	2

^b^: Free translation from French

^c^: The number of times the barrier/facilitator appeared in full transcripts

**Table 6:** Barriers and facilitators (Theoretical Domains Framework) about impact on patient safety and health

Theoretical domain	Barrier/ Facilitator	Representative excerpts of quotations^b^	Frequencies^c^
**Knowledge**	Recognition and acquisition of a LAST (Facilitator)	“Better recognition of a LAST, available kit”	1
	Knowledge acquisition with the course (Facilitator)	“Yes, in the sense that I may be favoring one imagery over another in an effort to be more efficient with some of the relevant information conveyed during the presentations”	1
	Knowledge improvement (Facilitator)	“Obtaining better knowledge”	1
**Skills**	Application of acquired competence (Facilitator)	“Better diagnostic accuracy”	1
	Reinforced competence (Facilitator)	“Better use and specific indications for PRP [for] gonarthrosis”	2
	Improved diagnostic capacity (Facilitator)	“Better recognition of a LAST, available kit”	1
	Better prescription of tests (Facilitator)	“Reduction of ordered tests”	1
	Improved test prescription (Facilitator)	“Yes, in the sense that I may be favouring one imaging over another for greater efficiency with some relevant information conveyed in presentations”	1
	Improved patient management (Facilitator)	“Better management”	3
	Improved assessment (Facilitator)	“More focused examination”	1
	New approach to prescribing treatment (Facilitator)	“Different way of prescribing opioid in outpatient…”	1
	Better diagnosis (Facilitator)	“At least 1 patient found to have coronary artery disease possibly secondary to radiation therapy as I am now aware that thoracic radiation therapy is a risk factor for coronary artery disease”	3
	Better prescribing (Facilitator)	“Better choice of neuraxial vs GA and pre-op analgesia medication”	1
**Social/Professional Role And Identity**	Better collaboration with another specialty (Facilitator)	“Better communication with radiology equals more accurate and targeted diagnosis …”	2
**Beliefs About Capabilities**	Confidence in ability (Facilitator)	“I feel better prepared to deal with patients with eating disorders”	1
	Enhanced competence (Facilitator)	“By confirming my way of practice”	1
	Better patient management (Facilitator)	“Earlier management of comorbidities”	5
	Better patient follow-up (Facilitator)	“More knowledge and therefore more adequate monitoring”	1
	Validation of an already well known practice (Facilitator)	“I already do it”	1
	Already aware of the practice (Facilitator)	“I was already aware of cardio safety in my practice”	1
**Beliefs About Consequences**	Application of practice guide (Facilitator)	“Cardiological follow-up”	1
	Better adapted treatment (Facilitator)	“Yes, a more precise diagnosis led to a better adapted treatment”	1
	Better prognosis with the adoption of a new approach to patient management (Facilitator)	“My patients for whom I would have been reluctant could benefit more from the medication and control their ADHD”	1
	Increased patient confidence (Facilitator)	“Definitely. They are more confident”	1
	No obvious impact (Barrier)	“Not known”	1
**Reinforcement**	Better management (Facilitator)	“Better outcome”	1
	Better prevention of complications (Facilitator)	“Prevention of complications”	1
**Memory, Attention And** **Decision Processes**	More attention to patient management (Facilitator)	“Because I paid more attention to the writing of the files among other things”	1
**Environmental Context And** **Resources**	Not enough patients to implement (Barrier)	“Few patients met, all without food problems”	1
	Change in clientele from that covered by the course (Barrier)	“I no longer do onco-psychiatry”	1
	Provision of necessary resources (Facilitator)	“Better recognition of a LAST, kit available”	1
	Clientele is not concerned by these areas (Barrier)	“No relation to health or safety”	1
	Limited to the use of available material (Barrier)	“Use of saturometer in minor surgery”	2
	Behavior had already been adopted before course (Barrier)	“I am already doing this”	1
	Already aware of the practice (Barrier)	“I was already aware of cardio safety in my practice”	1
**Behavioral Regulation**	Unsuitable clientele (Barrier)	“I no longer see patients in consultation”	1
	Better prognosis with the adoption of a new approach to patient management (Facilitator)	“My patients in whom I would have been reluctant could better benefit from the medication and control their ADHD”	1
	Adopted a new management approach (Facilitator)	“More patients treated who would not have been treated before”	1
	Adopted a new approach to follow-up (Facilitator)	“Change to my method of tracking test results”	1
	Increased family involvement in the patient care process (Facilitator)	“Involving families earlier in hospitalization”	1
^b^: Free translation from French ^c^: The number of times the barrier/facilitator appeared in full transcripts			

## Discussion

We evaluated the impact of a series of CPD courses given at an annual meeting of Québec specialist physicians by identifying factors influencing their intention to adopt targeted behaviors and assessing adoption of the behavior six months later. Overall, the increase in intention was statistically significant. Before courses, intention varied significantly across courses, but post-course this variance was not significant. ICCs showed that intention scores within course cohorts were more homogenous before courses than afterwards. Factors influencing increased intention post-course were moral norm, beliefs about capabilities and beliefs about consequences, with sensitivity analysis showing that moral norm was most significantly associated with increased intention. Association between intention post-courses and self-reported behavior change at six months after courses was significant. Barriers to adopting behaviors mostly related to environmental context and resources. These results bring us to make the following observations.

First, physicians’ intention to adopt a behavior improved significantly after courses when all courses were analyzed together, but when courses were analyzed individually, improvement varied across courses. This finding is consistent with published data [4, 40–42]. As the courses were different, we also compared means of change in intention among courses and found it increased significantly only for the sports injuries course. The sample size of this course (24 participants) may not, however, have been sufficient to detect a significant difference [43]. Across courses, intention scores varied significantly, but this variation was not significant post-course. Flint et al. propose that non-significant ANOVA tests suggest that cohorts are equal [44]. Similarly, our findings suggest that post-course, cohort intention scores improved and levelled out across courses. Therefore, our ICC estimates can be used for devising sample sizes for future trials.

Second, factors influencing physicians’ intention to adopt the targeted behaviors post-course were moral norm (its ethical acceptability), beliefs about capabilities (confidence about adopting the behavior) and beliefs about consequences (perception that the behavior would be useful and beneficial), but not social influences (perception about approval of their important people). Similar results were reported in another study about physicians’ intentions following CPD courses [18]. On the other hand, studies that combine several categories of health professional show that social influence seems to be an important influencing factor of participants’ intention [15, 19]. Some authors have reported a stronger effect of beliefs about capabilities [18, 19]. However, our results suggest that moral norm is the variable that most strongly influences intention after CPD courses. Our sensitivity analyses with participants who completed the CPD-REACTION both before and after the courses as well as sensitivity analyses with all participants except those who attended two CPD courses (and had two intention scores after courses), showed similar findings. Although the results of these sensitivity analyses were limited to two out of three factors in our final model, these results do not deviate far from the findings of our main analyses. Although there is no clear definition of when a given intention score is high enough to result in the adoption of a behavior, in light of our results, course design should consider these three modifiable psychosocial factors to increase the likelihood of adoption of a targeted behavior. Regarding moral norm, courses could raise participants’ awareness of the needs of others and present examples of behavior that reflects this in daily clinical practice [45]. In addition, courses could remind participants of the deontological dimension at the core of medical practice. Courses could also invite participants to argue for the targeted behavior, even if it seems abstract, in order to make it their own [45, 46]. As beliefs about capabilities directly modify actions and motivation and play a fundamental role in self-regulation and self-evaluation [47], CPD courses will need to give participants confidence in their abilities, promote feedback and provide reinforcing practical exercises [30, 48, 49]. Concerning beliefs about consequences, courses could provide information about the benefits of the behavior and personalized information about possible consequences for their practices and their patients [46]. Finally, bringing the patient voice into courses would be an effective way to remind physicians of the importance of CPD for their patients’ health [50, 51].

Third, we found a statistically significant association between intention after courses and self-reported behavior change at six months. Intention was significantly higher in participants who reported behavior change. This finding supports the argument that intention is a proxy of behavior [15]. Although direct measures of practice change would be more robust, they are difficult to achieve in pragmatic studies. However, intention after courses was relatively high (score ≥ 6.00) in both groups, a finding that limits the generalizability of this finding to all participants [52]. Our qualitative findings suggest that practice environment could have played an important role in the intention-behavior gap. Our participants mentioned that lack of resources and overloaded work schedules, for example, prevented them from adopting the behavior. In addition, in Québec there is a shortage of physicians, putting added pressure on them [53]. Despite these constraints, Godin suggests that intention based on moral norm may help to close the intention-behavior gap by keeping active the internal motivation to adopt a behavior [45]. Our respondents’ reports of impacts of the CPD course on their patients’ safety and health outcomes could be another rich source of motivation, showing the difference their adopting the behavior had made. In addition, CPD courses could attempt to close the intention-behavior gap with such methods as audit and feedback, “if-then” plans, commitment to change statements, or other methods of support for clinicians to follow through on their intentions [54, 55].

Our study has some limitations. First, there may have been a desirability bias. Second, from a theoretical point of view, there is some debate about whether one can derive meaningful results from diverse courses on different behaviors. However, due to the pragmatic nature of CPD course evaluation, the very large number of very diverse clinical topics and hence behaviors, and the small sample size of participants at the individual course level, we pooled all courses to compare intention before and after CPD courses. This gave us a sufficient sample size to detect a significant difference. This approach provided a macroscopic assessment of the impact of courses provided at the FMSQ’s annual meeting, while our qualitative results supplied a more microscopic focus. The most significant gain in intention occurred after a course that lasted 8 h instead of the more frequent 4 h. It is possible that this gain in intention was due to having spent more time in the course, although another course lasting 8 h showed no significant gain. The possible association of gain in intention with course duration is worth investigating further. Factors we identified will help CPD developers adjust their programs to have a greater impact on behavioral intention and hence on practice and patient outcomes. Third, although we found a significant association between intention post-course and self-reported behavior at six months, only a small proportion of our respondents provided this information. The low response rate may indicate a selection bias. This bias could underestimate the results and prevents us from generalizing them to the full group of specialist physicians. However, after quantitative sensitivity analyses our results were quite similar. In addition, this association is confirmed in other studies [22] and is coherent with the TPB [56]. While aggregating data on separate behaviors is not always advisable, this limitation was mitigated by respect for the theory archetypes that framed our study. In addition, the nine courses all targeted the adoption of behaviors corresponding with generic CanMED roles. ANOVA variance analyses showed that post-course intention did not vary significantly across the different courses. There could also be a social desirability bias related to behavior since we used self-reported questionnaires. Indeed, participants who did not complete the questionnaire sent before the courses were reminded to do so a few minutes before each course. This bias could produce an overestimate of the results. However, the fact that participation in our study was completely anonymous probably reduced the pressure of social desirability [57] and so we do not believe that it had a major impact on or calls into question the validity of the estimates obtained. To the best of our knowledge, this is the first theory-informed study to assess physicians’ intention and its influential factors before and after CPD courses at a congress using a validated measurement tool with follow-up at six months. These results contribute new knowledge about the real impact of medical CPD courses on physicians’ practice as well as contributing to the ongoing discussion about the intention-behavior gap. Implementing better strategies to encourage participation and reduce loss to follow-up would increase sample size and ensure better power for subgroup analyses and generalization of results. Further studies should explore factors relating to the practice environment, identify further barriers to adopting targeted behavior changes and develop strategies to circumvent them.

## Conclusion

Evidence from this study will equip CPD providers to improve the impact of their CPD courses. Moral norm, beliefs about capabilities and beliefs about consequences partially explain the intention to adopt a behavior. Investing in these modifiable factors could induce behavior change that will improve the health and safety of patients. CPD courses should be evaluated using a validated and reliable tool such as the CPD-REACTION questionnaire.

**Table:** 

**Lessons for Practice** • To increase the ethical acceptability (moral norm) of behaviors targeted by CPD courses, they could raise participants’ awareness of the needs of others (patients) using examples and role-play exercises demonstrating this in daily clinical practice. • To increase confidence about adopting a targeted behavior (beliefs about capabilities), course facilitators could check that participants feel equipped with the skills they need, promote feedback and provide reinforcing practical exercises. • To convince participants that the behavior is useful and beneficial (beliefs about consequences), courses could provide information about the benefits of the behavior and personalized information about the consequences.	

### Electronic supplementary material

Below is the link to the electronic supplementary material.

Supplementary Material 1

Supplementary Material 2

Supplementary Material 3

Supplementary Material 4

## Data Availability

The datasets used and/or analyzed during this study are available from the corresponding author on reasonable request.

## Declarations

### Previous presentations

- 50th annual meeting of the North American Primary Care Research Group, November 18 to 22, 2022 – USA.
- Congrès International d’Épidémiologie 2022, August 18 to 20, 2022 – Canada.

## List of Abbreviations

CPD: Continuing professional development
CI: Confidence interval
SD: Standard deviation
ICC: Intraclass correlation coefficient
FMSQ: Fédération des médecins spécialistes du Québec
TDF: Theoretical Domain Framework
