# Supplementary material for: Evaluating the impact of continuing professional development courses on physician behavioral intention: a pre-post study with follow-up at six months
Source: BMC Med Educ. 2023 Sep 3;23:629. doi: 10.1186/s12909-023-04597-3 (PMC10476392; doi:10.1186/s12909-023-04597-3)
Supplement: Supplementary file 1 — Supplementary Material 1 [file 12909_2023_4597_MOESM1_ESM.pdf]

Appendix 1 Selected CPD courses and their main targeted behaviors

	CPD Course	Duration	Main behavior
1	Essentials of Patient Safety Education and Assessment (Patient Safety)	4 hours	Teach my learners the principles of patient safety and continuous improvement in the hospital setting
2	A care incident is more than just a mishap! (Care incident)	4 hours	Complete an incident/accident report according to the guidelines in effect in my institution when a care incident occurs
3	The ERC initiative: an innovative interdisciplinary approach to optimizing care (Optimizing care)	4 hours	Use at least one of the 6 recommendations of Optimized Recovery Canada when managing perioperative patients
4	Can the opioid crisis be contained through optimal perioperative pain management? (Perioperative pain-Opioid)	4 hours	Prescribe opioids in a safe manner when managing perioperative pain in my patients
5	Sports injuries: assessment and treatment of common musculoskeletal conditions (Sports injuries)	8 hours	Use a clinician-radiologist approach to investigate common musculoskeletal injuries in active patients
6	Eating disorders in adolescents (Eating disorders)	8 hours	Systematically assess the medical and psychological health status of my patients with eating disorders
7	ADHD is also a matter of the heart (Attention deficit)	4 hours	To use psychostimulants in a safe manner for ADHD patients with heart problems
8	Cardio-oncology, to get to the bottom of it! (Cardio-oncology)	4 hours	Use a cardiologist-hemato-oncologist approach to manage cardiovascular risks induced by hemato-oncology in my patients
9	Local anesthesia: when blocking rhymes with toxicity (Local anesthesia)	4 hours	Follow the 2018 ASRA recommendations for the management of systemic toxicity resulting from local anesthesia (LAST kit)
