# Supplementary material for: Evaluating the impact of continuing professional development courses on physician behavioral intention: a pre-post study with follow-up at six months
Source: BMC Med Educ. 2023 Sep 3;23:629. doi: 10.1186/s12909-023-04597-3 (PMC10476392; doi:10.1186/s12909-023-04597-3)
Supplement: Supplementary file 2 — Supplementary Material 2 [file 12909_2023_4597_MOESM2_ESM.pdf]

Appendix 2 CPD-REACTION questionnaire constructs: mean scores and intraclass correlation coefficient (ICC) before CPD courses

CPD-REACTION Constructs	CPD Courses														
	Patient Safety			Healthcare Incident			Optimization of Care			Perioperative Pain			Sports Injuries		
**Score range (1-7)**	**n**	**Mean (SD)**	**Median (Interquartile Range)**	**n**	**Mean (SD)**	**Median (Interquartile Range)**	**n**	**Mean (SD)**	**Median (Interquartile Range)**	**n**	**Mean (SD)**	**Median (Interquartile Range)**	**n**	**Mean (SD)**	**Median (Interquartile Range)**
**Intention**	9	6.00 (1.20)	6.50 (5.00 ; 7.00)	9	5.94 (1.49)	6.50 (5.50 ; 7.00)	6	5.75 (1.08)	5.50 (5.00 ; 7.00)	13	6.23 (1.48)	7.00 (6.50 ; 7.00)	45	5.59 (1.33)	6.00 (5.00 ; 7.00)
**Beliefs about capabilities**	9	5.13 (1.36)	5.00 (4.00 ; 6.50)	9	5.67 (1.37)	6.00 (5.33 ; 6.33)	6	5.06 (1.27)	4.50 (4.00 ; 6.67)	13	5.49 (0.92)	5.67 (5.00 ; 6.00)	45	5.26 (1.38)	5.33 (4.67 ; 6.33)
**Social influences**	9	2.93 (1.15)	3.00 (2.00 ; 3.67)	9	4.22 (1.38)	5.00 (3.67 ; 5.00)	6	3.94 (1.53)	4.00 (2.33 ; 4.67)	13	4.85 (0.99)	5.33 (4.67 ; 5.33)	45	4.19 (1.29)	4.33 (3.33 ; 5.33)
**Moral norm**	9	6.67 (0.56)	7.00 (6.50 ; 7.00)	9	6.33 (0.56)	6.50 (6.00 ; 6.50)	6	5.67 (1.25)	5.75 (4.50 ; 7.00)	13	6.23 (1.15)	6.50 (6.00 ; 7.00)	45	5.99 (0.97)	6.00 (5.50 ; 7.00)
**Beliefs about consequences**	9	6.17 (1.41)	7.00 (6.00 ; 7.00)	9	5.17 (1.06)	5.00 (4.50 ; 5.50)	6	5.42 (1.80)	6.00 (5.00 ; 6.50)	13	6.04 (1.39)	7.00 (6.00 ; 7.00)	45	5.83 (1.25)	6.00 (5.00 ; 7.00)

Abbreviations: CPD indicates continuing professional development; SD indicates Standard deviation

Appendix 2 CPD-REACTION questionnaire constructs: mean scores and intraclass correlation coefficient (ICC) before CPD courses **(Continued)**

**CPD-REACTION Constructs**	**CPD Courses**										
	**Eating Disorders**		**Attention Deficit and Hyperactivity Disorder**		**Cardio-Oncology**		**Local Anesthesia**		**All CPD Activities**		**ICC**
**Score range (1-7)**	**n Mean (SD)**	**Median (Interquartile Range)**	**n Mean (SD)**	**Median (Interquartile Range)**	**n Mean (SD)**	**Median (Interquartile Range)**	**n Mean (SD)**	**Median (Interquartile Range)**	**n Mean (SD)**	**Median (Interquartile Range)**	
**Intention**	15 6.13 (1.13)	6.50 (6.00 ; 7.00)	20 4.30 (2.07)	4.75 (2.25 ; 6.00)	17 5.97 (1.04)	6.50 (5.50 ; 6.50)	23 6.41 (1.37)	7.00 (7.00 ; 7.00)	157 5.74 (1.51)	6.00 (5.00 ; 7.00)	0.14
**Beliefs about capabilities**	15 5.11 (1.09)	5.33 (4.00 ; 5.67)	20 3.67 (1.65)	3.83 (2.17 ; 4.83)	17 4.76 (1.32)	5.00 (4.00 ; 5.33)	23 5.52 (1.48)	6.00 (5.33 ; 6.33)	157 5.05 (1.45)	5.33 (4.33 ; 6.00)	0.13
**Social influences**	15 4.27 (1.20)	4.33 (3.33 ; 5.00)	20 3.12 (1.22)	3.00 (2.00 ; 4.00)	18 4.33 (1.11)	4.67 (3.33 ; 5.33)	23 4.46 (1.47)	4.67 (4.33 ; 5.33)	158 4.09 (1.35)	4.33 (3.00 ; 5.33)	0.14
**Moral norm**	15 6.10 (0.83)	6.00 (5.50 ; 7.00)	20 4.48 (1.59)	4.50 (3.75 ; 5.75)	18 6.25 (0.79)	6.00 (6.00 ; 7.00)	23 6.61 (0.93)	7.00 (7.00 ; 7.00)	158 5.99 (1.18)	6.00 (5.50 ; 7.00)	0.27
**Beliefs about consequences**	15 6.37 (0.64)	6.50 (6.00 ; 7.00)	20 4.95 (1.45)	5.00 (4.00 ; 6.00)	18 6.17 (1.21)	7.00 (6.00 ; 7.00)	23 6.63 (0.84)	7.00 (6.50 ; 7.00)	158 5.91 (1.29)	6.00 (5.00 ; 7.00)	0.14

Abbreviations: CPD indicates continuing professional development; SD indicates Standard deviation
