# Supplementary material for: Evaluating the impact of continuing professional development courses on physician behavioral intention: a pre-post study with follow-up at six months
Source: BMC Med Educ. 2023 Sep 3;23:629. doi: 10.1186/s12909-023-04597-3 (PMC10476392; doi:10.1186/s12909-023-04597-3)
Supplement: Supplementary file 3 — Supplementary Material 3 [file 12909_2023_4597_MOESM3_ESM.pdf]

Appendix 3 CPD-REACTION questionnaire constructs: mean scores and intraclass correlation coefficient (ICC) after CPD courses

**CPD-REACTION Constructs**	**CPD Courses**														
	**Patient Safety**			**Healthcare Incident**			**Optimization of Care**			**Perioperative Pain**			**Sports Injuries**		
**Score range (1-7)**	**n**	**Mean (SD)**	**Median (Interquartile Range)**	**n**	**Mean (SD)**	**Median (Interquartile Range)**	**n**	**Mean (SD)**	**Median (Interquartile Range)**	**n**	**Mean (SD)**	**Median (Interquartile Range)**	**n**	**Mean (SD)**	**Median (Interquartile Range)**
**Intention**	6	6.75 (0.42)	7.00 (6.50 ; 7.00)	7	6.71 (0.49)	7.00 (6.00 ; 7.00)	9	6.22 (0.94)	6.50 (6.00 ; 7.00)	8	6.50 (0.60)	6.75 (6.00 ; 7.00)	39	6.13 (1.00)	6.00 (6.00 ; 7.00)
**Beliefs about capabilities**	6	5.67 (1.19)	5.83 (4.67;6.67)	7	6.10 (0.76)	6.33 (5.33;6.67)	9	5.56 (1.58)	6.00 (5.33;6.67)	8	6.08 (0.90)	6.17 (5.67;6.83)	39	5.88 (1.01)	6.00 (5.67;6.67)
**Social influences**	6	3.28 (1.57)	3.50 (2.00 ; 4.33)	7	3.48 (1.69)	3.67 (2.00 ; 5.00)	9	4.30 (1.24)	4.67 (3.33 ; 5.00)	8	5.17 (0.76)	5.33 (4.33 ; 5.83)	39	4.74 (1.14)	5.00 (4.33 ; 5.67)
**Moral norm**	6	6.92 (0.20)	7.00 (7.00;7.00)	7	6.79 (0.39)	7.00 (6.50;7.00)	9	6.33 (1.03)	7.00 (6.00;7.00)	8	6.38 (1.06)	7.00 (6.00;7.00)	38	6.20 (0.96)	6.00 (6.00;7.00)
**Beliefs about consequences**	6	6.92 (0.20)	7.00 (7.00;7.00)	7	5.64 (1.31)	6.00 (4.00;7.00)	9	6.11 (1.17)	7.00 (5.00;7.00)	8	6.63 (0.52)	7.00 (6.00;7.00)	39	6.06 (1.03)	6.00 (5.50;7.00)

Abbreviations: CPD indicates continuing professional development; SD indicates standard deviation

Appendix 3 CPD-REACTION questionnaire constructs: mean scores and intraclass correlation coefficient (ICC) after CPD courses **(continued)**

**CPD-REACTION Constructs**	**CPD Courses**															
	**Eating Disorders**			**Attention Deficit and Hyperactivity Disorder**			**Cardio-Oncology**			**Local Anesthesia**			**All CPD Activities**			**ICC**
**Score range (1-7)**	**n**	**Mean (SD)**	**Median (Interquartile Range)**	**n**	**Mean (SD)**	**Median (Interquartile Range)**	**n**	**Mean (SD)**	**Median (Interquartile Range)**	**n**	**Mean (SD)**	**Median (Interquartile Range)**	**n**	**Mean (SD)**	**Median (Interquartile Range)**	
**Intention**	16	6.56 (0.83)	7.00 (6.25 ; 7.00)	9	6.22 (1.46)	7.00 (6.00 ; 7.00)	17	6.38 (0.57)	6.50 (6.00 ; 7.00)	18	6.42 (1.15)	7.00 (6.50 ; 7.00)	129	6.35 (0.93)	7.00 (6.00 ; 7.00)	0.004
**Beliefs about capabilities**	16	5.96 (0.70)	6.00 (5.67 ; 6.33)	9	6.07 (1.16)	6.33 (5.67 ; 7.00)	17	5.45 (1.10)	5.67 (5.00 ; 6.33)	18	6.33 (0.79)	6.67 (6.33 ; 7.00)	129	5.90 (1.02)	6.00 (5.67 ; 6.67)	0.02
**Social influences**	16	4.67 (1.05)	5.00 (4.17 ; 5.33)	9	4.63 (1.14)	4.33 (4.33 ; 5.33)	17	4.78 (0.89)	5.00 (4.67 ; 5.33)	18	4.91 (1.15)	5.17 (4.33 ; 6.00)	129	4.61 (1.20)	5.00 (4.00 ; 5.33)	0.11
**Moral norm**	16	6.47 (0.88)	7.00 (6.00 ; 7.00)	9	6.44 (1.01)	7.00 (6.00 ; 7.00)	17	6.74 (0.44)	7.00 (6.50 ; 7.00)	17	6.71 (0.79)	7.00 (7.00 ; 7.00)	127	6.48 (0.85)	7.00 (6.00 ; 7.00)	0.03
**Beliefs about consequences**	16	6.41 (0.92)	7.00 (6.00 ; 7.00)	9	6.06 (1.63)	7.00 (6.00 ; 7.00)	17	6.56 (0.53)	7.00 (6.00 ; 7.00)	18	6.64 (0.74)	7.00 (6.50 ; 7.00)	129	6.31 (0.98)	7.00 (6.00 ; 7.00)	0.04

Abbreviations: CPD indicates continuing professional development; SD indicates Standard deviation
