# Supplementary material for: Evaluating the impact of continuing professional development courses on physician behavioral intention: a pre-post study with follow-up at six months
Source: BMC Med Educ. 2023 Sep 3;23:629. doi: 10.1186/s12909-023-04597-3 (PMC10476392; doi:10.1186/s12909-023-04597-3)
Supplement: Supplementary file 4 — Supplementary Material 4 [file 12909_2023_4597_MOESM4_ESM.docx]

**Electronic supplementary material**

Additional file 1 (pdf): Appendix 1 Selected CPD courses and their main behaviors

Description of data: This table presents the full title, the duration and targeted behavior of each of nine CPD Courses included in this study

Additional file 2 (pdf): Appendix 2 CPD-REACTION questionnaire constructs mean scores and intraclass correlation coefficient (ICC) before CPD courses.

Description of data: This table presents for each course, intention scores and psychosocial factors that influence intention scores before courses and their ICC

Additional file 3 (pdf): Appendix 3 CPD-REACTION questionnaire constructs mean scores and intraclass correlation coefficient (ICC) after CPD courses

Description of data: This table presents for each course, intention scores and psychosocial factors that influence intention scores after courses and their ICC
